# How family support alleviates death anxiety in breast cancer patients: the mediating role of meaning in life

**DOI:** 10.3389/fpubh.2025.1567485

**Published:** 2025-04-01

**Authors:** Gui Gui, Dajun Yang, Yujie Liu, Yisong Yao, Xinling Xie, Ruining Liu, Mingye Liu, Heming Liu, Fangfang Zhou

**Affiliations:** ^1^Institute of Basic Medicine, North Sichuan Medical College, Nanchong, China; ^2^School of Clinical Medicine, North Sichuan Medical College, Nanchong, China; ^3^Sichuan Provincial Primary Health Service Development Research Center, North Sichuan Medical College, Nanchong, China; ^4^Key Laboratory of Digital-Intelligent Disease Surveillance and Health Governance, North Sichuan Medical College, Nanchong, China; ^5^School of Administration, North Sichuan Medical College, Nanchong, China; ^6^School of Nursing, North Sichuan Medical College, Nanchong, China

**Keywords:** family support, death anxiety, meaning in life, breast cancer patients, meaning therapy theory

## Abstract

Previous studies have predominantly focused on the relationship between death anxiety and quality of life in breast cancer patients, with limited exploration on how to alleviate their death anxiety. To address this gap, we recruited 533 breast cancer patients and utilized structural equation modeling and Process Model 4 to analyze the internal mechanisms and boundary conditions between family support and death anxiety. The study results indicated that family support significantly negatively impacts death anxiety in breast cancer patients; similarly, meaning in life also significantly negatively impacts death anxiety. More importantly, we found that meaning in life plays a full mediating role between family support and death anxiety. This study suggests that by enhancing family support levels and strengthening patients’ perception of meaning in life, we can significantly improve the psychological health status of breast cancer patients, thereby potentially improving their quality of life.

## 1 Introduction

Breast cancer, originating from the epithelium of the breast ducts or lobules (1, 2), is one of the most common malignant tumors in women globally (3). According to global disease burden statistics, the incidence of breast cancer increased by 128% from 1990 to 2019, becoming one of the most severe threats to women’s health worldwide (4). With continuous advancements in medical technology, the main clinical treatments for breast cancer include surgery, chemotherapy, and endocrine therapy (5). Despite significant progress in modern medical means, the risk of breast cancer recurrence remains high (6, 7). Moreover, a considerable proportion of breast cancer patients often experience anxiety due to the uncertainty of treatment outcomes and fear of recurrence and metastasis, harboring concerns about subsequent treatment measures (8). When facing death, breast cancer patients are prone to feelings of anxiety, fear, and pessimism (9). In the field of psychology, this negative psychological state related to death is termed death anxiety (10). It refers to the distress experienced when an individual is confronted with the threat of death, including worries about the afterlife, the dying process, and the non-existence of the self (11). Death anxiety is a common psychological issue among cancer patients (12). It can severely impede the physical and psychological recovery of cancer patients and may even accelerate their progression towards death (13). This anxiety intensifies when people realize that death is inevitable, especially when facing a life-threatening illness that has reached an incurable stage (14). In recent years, research has gradually focused on the significant impact of family and social support on the emotional well-being and quality of life of breast cancer patients (15, 16). Social support has been proven to be an effective buffer in alleviating anxiety and depression in breast cancer patients (17, 18). However, this study has not further discussed the relationship between family support and death anxiety. Currently, research on the relationship between family support and death anxiety in breast cancer patients is still relatively limited. Therefore, conducting a study on the impact of family support on death anxiety in breast cancer patients is of great significance.

Family support provides care, assistance, and encouragement to family members in various aspects such as psychology, materials, and emotional support (19–21). In recent years, researchers have increasingly emphasized the positive role of family support in the recovery of cancer patients (22). For example, emotional comfort and material support in family interactions have been proven to help alleviate death anxiety in breast cancer patients (23). However, previous studies have mainly focused on areas such as patients’ self-management behaviors, post-traumatic psychological resilience, and family adaptation (24–26), with few studies exploring the direct connection between family support and death anxiety. Specifically, family support significantly enhances the psychological healing ability of breast cancer patients by providing emotional warmth, a sense of belonging, and the courage to face death and illness. This support is crucial for improving patients’ psychological resilience and their ability to cope with disease challenges. Therefore, this study attempts to answer: Does family support have a positive impact on death anxiety in breast cancer patients? What are the internal mechanisms between family support and death anxiety in breast cancer patients?

According to logotherapy, meaning in life is not only a psychological motivation that drives individuals to pursue personal values but also an important means applied in positive psychotherapy (27, 28). Previous studies have shown that enhancing the sense of meaning in life is a key measure to improve the psychological status of critically ill patients and alleviate death anxiety (29, 30). For breast cancer patients, a higher sense of meaning in life is significantly correlated with greater individual happiness and quality of life (31), as well as fewer negative anxiety emotions and a stronger desire to survive, and patients exhibit good treatment compliance (32). However, although previous studies have revealed a strong correlation between meaning in life and death anxiety, the relationship between the two has not been further explored in the context of family support. Family support is considered an important way for breast cancer patients to enhance their sense of meaning in life (33, 34). A positive attitude and emotional support within the family structure can enhance patients’ sense of meaning in life, thereby reducing their anxiety levels (35). At the same time, the perception of life meaning and the pursuit of life values help breast cancer patients increase their positive psychological emotions and alleviate death anxiety (36). A study in Powers et al. (37) has confirmed that family support can effectively improve patients’ treatment confidence and relieve fear emotions. Logotherapy theory emphasizes that an individual’s ability to find meaning in difficult situations is central to their mental health (38, 39). For breast cancer patients, family support not only provides material help, but also helps patients find new life goals and meaning during the disease process through family members’ daily companionship, listening and encouragement (40, 41). For example, family members can help patients reassess the value of life and enhance their sense of hope for the future by sharing the patient’s treatment progress and making rehabilitation plans together (42). Therefore, we hypothesize that meaning in life plays a synergistic mediating role in the alleviation of death anxiety by family support, and the two jointly act to improve patients’ psychological health and alleviate patients’ death anxiety emotions.

The family support model emphasizes the important role of family members in the recovery process of patients, including emotional support, information support, and practical help (43). In this study, family support alleviated death anxiety by improving patients’ sense of meaning in life. This finding complements the family support model and further emphasizes the central role of the family in the psychological recovery of patients. Further, in the theory of family support, intergenerational support is considered an important aspect of family support (44), while meaning in life is regarded as a more positive coping mechanism. The enhancement and maintenance of the sense of meaning in life are considered an automatic defensive response to death anxiety, with the aim of transforming negative emotions about illness and stress into positive thoughts through positive psychological adjustment (45). This automatic response reflects a natural, often unconscious, psychological mechanism that helps individuals cope with existential threats by fostering a sense of purpose and coherence in life (46, 47). This logotherapy theory not only provides effective support for alleviating death anxiety but, more importantly, focuses on patients’ psychological needs, that is, the motivation to drive individuals to pursue personal values and enhance individuals’ profound understanding of their own existential value (48). The most effective way to protect oneself from death anxiety is to focus on enhancing the sense of meaning in life and family support. Breast cancer patients can alleviate death anxiety through increased family support and a strengthened sense of meaning in life. These improvements are often facilitated by external interventions, such as family-based support programs and meaning-centered therapies, may contribute to better quality of life.

The above relationship was verified again in the resilience theory. The theory holds that individuals are able to maintain or restore mental health in the face of adversity through the mobilization of internal and external resources (49, 50). For breast cancer patients, family support is an important external resource (51), which can help patients enhance their psychological resilience, so as to better cope with the psychological pressure brought by the disease. A sense of meaning in life, on the other hand, can be viewed as an internal resource that enhances patients’ ability to cope with illness by helping them find purpose and value in life, thereby reducing death anxiety (52).

Based on the above analysis, we propose three hypotheses:

*H1:* Family support has a significant negative impact on death anxiety in breast cancer patients.

*H2:* Meaning in life has a significant negative impact on death anxiety in breast cancer patients.

*H3:* Meaning in life plays a significant mediating role between family support and death anxiety in breast cancer patients.

## 2 Materials and methods

### 2.1 Sample

This study aims to examine how family support alleviates death anxiety in breast cancer patients. Therefore, the inclusion criteria for our target participants were: (1) clinically diagnosed with breast cancer; (2) having normal verbal communication ability; and (3) no significant cognitive impairment. Among them, we used the Mini-Mental State Examination Scale to test cognitive impairment, with a total score below 23 points as the standard (53).

This study was approved by the Academic Ethics Committee of North Sichuan Medical College and conducted data collection and recruitment at the Affiliated Hospital of North Sichuan Medical College. All participants signed an electronic informed consent form, which was explained in the approval document of the Academic Ethics Committee of North Sichuan Medical College. Since this study did not involve patients under 18 years old, there was no need to provide parental informed consent.

The sample size was analyzed by G * Power 3.1.9.7 software. Referring to the study of Lee et al. (54), we set the F-test as “Linear multiple regression: fixed model, R2 deviation from zero.” The effect size was set as f^2^ = 0.15, significance level *α* = 0.05, statistical power (1−*β*) = 0.8, and the total number of required samples was calculated as 68. The data collection period of this study was from August to September 2024, with a total of 550 questionnaires distributed. Participant recruitment process: We first contacted the doctors in the breast department of the Affiliated Hospital of North Sichuan Medical College, and explained the purpose and content of the study with the breast doctors, and stored the two-dimensional code of the electronic questionnaire and paper questionnaire in the breast department. When a breast cancer patient visited the clinic, we briefly introduced the purpose and content of the study to the patient, and collected the questionnaire data after obtaining the consent of the patient. The electronic questionnaire could not be submitted without completed responses (incomplete questionnaires) during the collection process. In the process of paper questionnaire, the questionnaires with missing data were eliminated in the data cleaning stage to enhance the accuracy of the data. Excluding questionnaires with strong consistency in answers and short answering time (less than 2 min), 17 samples were removed, and the effective rate was 96.9%. The standard of answering time less than 2 min comes from the research of Kost and da Rosa (55). Meanwhile, the length and question type of the questionnaire will affect the answering time of the questionnaire Liu and Wronski (56). In this study, there are only 38 questions in total, the actual length of the questionnaire is relatively long, the measurement questions are in the form of a scale, the length of the questions is longer, and the response time should be more than 2 min. Strong consistency in answers means that the specific answer agreement of questionnaire responses is higher than 95%. This standard is referred to the research of Huang et al. (57) and Meade and Craig (58). For specific demographic information, see Table 1.

**Table 1:** Summary of demographic information.

Variable	Option	Number	Proportion
Gender	Male	49	9.2%
	Female	484	90.8%
Age	18–25 years old	13	2.4%
	26–40 years old	103	19.3%
	41–60 years old	186	34.9%
	61–70 years old	146	27.4%
	71 years old and above	85	15.9%
Educational background	Primary school	15	2.8%
	Middle school	124	23.2%
	Secondary specialized school, high school	194	36.5%
	Junior college	104	19.5%
	Undergraduate college	65	12.2%
	Postgraduate	31	5.8%
Habitation	City	317	59.5%
	Countryside	216	40.5%

### 2.2 Research methods

#### 2.2.1 Family Support Scale

The measurement of family support was based on the family support section of the Perceived Social Support Scale (59), which assesses patients’ perceptions of support from family members. This section consists of 4 items, such as “My family can provide me with concrete and specific help” and “When I need it, I can get emotional help and support from my family.” We scored using a 5-point Likert scale. Higher scores indicate higher family support for patients. In this study, the Cronbach’s *α* of this scale was 0.845. AMOS 29.0 was used to construct a variable model and analyze the model fitting coefficient of the scale. According to the test standard proposed by Bagozzi and Yi (60), Scott and Bruce (61) and Hayduk (62), the family support model fitted well (CMID/DF = 2.925, GFI = 0.956, AGFI = 0.934,CFI = 0.981, TLI = 0.974, RMSEA = 0.05).

#### 2.2.2 Meaning in Life Scale

The Meaning in Life Scale was based on the research by Steger et al. (63), and our measurement items were adapted from two dimensions of this scale, totaling 15 measurement items. For example, “I understand the meaning of my life.” The two dimensions are the presence of meaning in life and the search for meaning in life. We scored using a 5-point Likert scale. Higher scores indicate higher meaning in life for patients. In this study, the Cronbach’s *α* of this scale was 0.912. According to the test criteria proposed by Bagozzi and Yi (60), Scott and Bruce (61), and Hayduk (62), the model of family support was well fitted (CMID/DF = 3.744, GFI = 0.953, AGFI = 0.929, CFI = 0.974, TLI = 0.966, RMSEA = 0.06).

#### 2.2.3 Death Anxiety Scale

The Death Anxiety Scale was based on the breast cancer fear scale developed by Templer (64). This scale includes 15 items, divided into four dimensions: emotion (6 items), stress and suffering (4 items), time awareness (2 items), and cognition (3 items). For example: “Do you agree that when you think about the serious consequences of breast cancer, do you feel scared?” We scored using a 5-point Likert scale. Higher scores indicate higher death anxiety for patients. In this study, the Cronbach’s *α* of this scale was 0.794. According to the test standard proposed by Bagozzi and Yi (60), Scott and Bruce (61), and Hayduk (62), the family support model fitted well (CMID/DF = 7.072, GFI = 0.976,AGFI = 0.904,CFI = 0.978, TLI = 0.93, RMSEA = 0.089).

#### 2.2.4 Cultural adaptability of the scale

The process of cultural adaptation. Firstly, in the process of designing the questionnaire, we used Chinese and English to design the questions. At the same time, in order to adapt to the Chinese cultural background, we consulted an expert review of the scale to ensure the accuracy and applicability of the measurement content. Finally, we read a large body of literature that applied the three scales to study the Chinese breast cancer population. This indicates that the scale performs well in the Chinese breast cancer patient population.

## 3 Results

### 3.1 Common method Bias

Referring to the study by Fuller et al. (65), we used the Harman single-factor analysis method to test for common method bias by conducting an exploratory factor analysis on all variables’ items. The results showed that without rotation, a total of 7 factors with eigenvalues greater than 1 appeared, and the explained variance of the first factor was 31.294%, which did not exceed the critical value of 40%, indicating that there was no serious common method bias in this study.

Then, referring to Podsakoff et al. (66) and Lindell and Whitney (67) proposal to use control variables to reduce the common method bias, we used the method of one-way analysis of variance, taking gender and age as control variables and death anxiety as dependent variable. The results showed that gender and death anxiety, age and death anxiety had no significant effect, and the specific experimental results are shown in Section 3.2. Therefore, the absence of common method bias in this study was again confirmed and the accuracy of the study was enhanced.

### 3.2 One-way ANOVA for demographic information

Gender and death anxiety. We performed a one-way ANOVA with sex as the independent variable and death anxiety as the dependent variable. The results showed that the gender difference of breast cancer patients had no significant effect on death anxiety (M _male_ = 3.19, SD _male_ = 0.257; M _female_ = 3.2, SD _female_ = 0.496, *F* (1,531) =0.037, *p* = 0.847). The reason for this phenomenon may be that men and women experience common psychosocial factors when facing breast cancer, such as physical defects caused by surgery, response to chemotherapy, and financial stress (68–70). The effect of these factors on death anxiety may have masked the role of gender differences. For example, surgical removal of the breast not only affects the physical beauty of women, but also has a negative impact on the self-image of male patients, resulting in greater psychological stress for both (71, 72). At the same time, breast cancer as a serious malignant tumor, its own characteristics and treatment process have a great impact on the psychological state of patients (73). Disease progression, treatment effect, recurrence risk and other factors have a direct and strong impact on the death anxiety of patients (74, 75). These factors were similarly represented in male and female patients, possibly making the role of gender differences in death anxiety relatively attenuated.

Age and death anxiety. We performed a one-way ANOVA with age as the independent variable and death anxiety as the dependent variable. The results showed that the age difference of breast cancer patients had no significant effect on death anxiety (*F* (4,528) = 0.348, *p* = 0.846). According to the cognitive evaluation theory (76), the cognitive evaluation of the threat and meaning of death can affect the level of death anxiety (77, 78). Breast cancer patients of different ages may have different cognition and evaluation of disease, but their influence on death anxiety is not necessarily significantly different (79). For example, both young and older breast cancer patients may view breast cancer as a serious life threatening disease and worry about future uncertainty and possible death, and this similarity in cognitive evaluation results in death anxiety that does not differ significantly between age groups. This finding is a valid response to (80).

Habitation and family support. We performed a one-way ANOVA with place of residence as the independent variable and family support as the dependent variable. The results showed that the residence of breast cancer patients had no significant effect on family support (*F* (1,531) = 0.966, *p* = 0.326). The place of residence of breast cancer patients may affect the specific living environment and resource access of the family (35), but the core aspects of family function, such as emotional support, decision-making ability, and communication style, do not differ significantly by the change of place of residence. A large number of studies have pointed out that the overall status of family function of cancer patients is relatively consistent in different regions, and family members can provide necessary support and help to a certain extent (81, 82).

### 3.3 Descriptive statistics of main variables

We conducted descriptive statistics on family support, meaning in life, and death anxiety, as shown in Table 2. We found that breast cancer patients had lower family support perception (M = 2.365, SD = 0.855) and higher death anxiety (M = 3.198, SD = 0.478). However, the meaning of life of breast cancer patients (M = 2.782, SD = 0.648) was higher than that of family support. Then, according to the normality test standard proposed by Kline (83): the absolute value of skewness coefficient is less than 3, and the absolute value of kurtosis coefficient is less than 8, the sample data can be regarded as meeting the requirements of approximately normal distribution. We found that the absolute values of the measured items of kurtosis and skewness for each variable in this study satisfied a normal distribution.

**Table 2:** Descriptive statistics of principal variables.

Variable	M	SD	Skewness	Kurtosis
Family support	2.365	0.855	0.603	0.94
Meaning in life	2.782	0.648	0.205	2.11
Death anxiety	3.198	0.478	0.507	4.03

### 3.4 Correlation analysis

We conducted correlation analysis on family support, meaning in life, and death anxiety. The results showed that family support was significantly negatively correlated with death anxiety (r = −0.352, *p* < 0.01); family support was significantly positively correlated with meaning in life (r = 0.561, *p* < 0.01); meaning in life and death anxiety were significantly negatively correlated (*r* = −0.421, *p* < 0.01). For specific details, see Table 3.

**Table 3:** Correlation analysis results of each variable.

Variable	1	2	3
1. Family support	1		
2. Meaning in life	0.561**	1	
3. Death anxiety	−0.352**	−0.421**	1

### 3.5 Mediating effect of meaning in life

**Figure 1:**
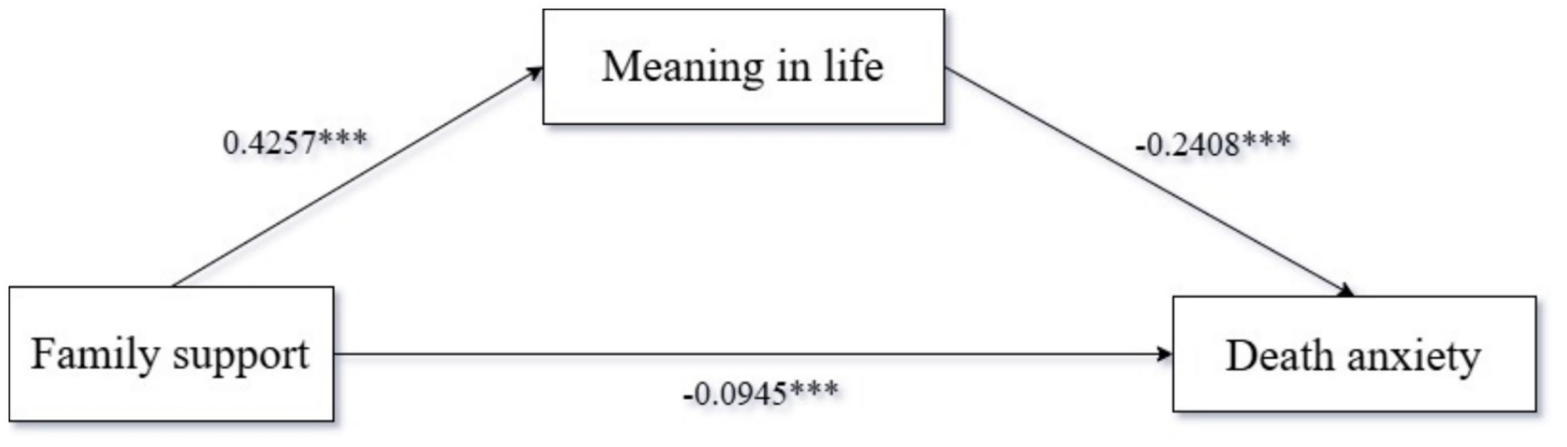
Path coefficient plot of mediating effects of meaning in life.

We tested the mediating effect of meaning in life, with family support as the independent variable and death anxiety as the dependent variable. We used Process Model 4 to analyze the mediating relationship of meaning in life between family support and death anxiety [Bootstrap sample: 5000; (84)]. The results showed that the mediating process of family support—meaning in life—death anxiety was significant (*β* = −0.1025, SE = 0.0269, 95%CI = [−0.1561, −0.0951]). Specifically, family support had a significant negative impact on death anxiety (*β* = −0.0945, *p* < 0.001, 95%CI = [−0.15, −0.04]); meaning in life had a significant negative impact on death anxiety (*β* = −0.2408, *p* < 0.001, 95%CI = [−0.3089, −0.1726]); and family support had a significant positive impact on meaning in life (*β* = 0.4257, *p* < 0.001, 95%CI = [0.37, 0.47]). Therefore, meaning in life fully mediated the relationship between family support and death anxiety, confirming Hypothesis H3. As shown in Table 4 and Figure 1.

**Table 4:** Table of regression coefficients for mediating effects of meaning in life.

Regression equation		Overall fit index			Significance of regression coefficient		
Outcome variables	Predictive variables	*R*	*R* ^2^	*F*	*β*	*t*	*95%CI*
Meaning in life	Family support	0.561	0.315	243.97***	0.4257	15.619***	[0.37, 0.47]
Death anxiety	Family support	0.443	0.197	65.018***	−0.0945	−3.5926***	[−0.15, −0.04]
	Meaning in life				−0.2408	−6.9428***	[−0.3089, −0.1726]

## 4 Discussion

### 4.1 Negative impact of family support on death anxiety

The study confirms that family support has a negative impact on death anxiety in breast cancer patients. Within the family system, when one member falls ill, other members coordinate to provide support for the breast cancer patient, thereby maintaining family balance (85, 86). This support is mainly manifested in two aspects: emotional support and material support (44, 87). In terms of emotional support, family members provide psychological comfort to the patient, alleviating their fear (88). In terms of material support, family members provide a good medical environment for the patient, ensuring the continuity of treatment (89). This dual support of material and emotional aspects not only enhances the psychological resilience and self-efficacy of breast cancer patients but also significantly improves treatment outcomes and quality of life. This study further explores the impact mechanism of death anxiety in breast cancer patients from a micro perspective, enriching the research on the antecedents of death anxiety. Previous studies have mostly discussed the mitigation effect of social support on patients’ death anxiety from a macro perspective (90). By revealing the specific pathways of family support, this study fills this research gap and provides a new perspective for clinical intervention. Moreover, this study also offers valuable guidance for patients’ families. When providing support, family members should focus on the combination of emotional and material support to enhance the patient’s psychological resilience and quality of life.

### 4.2 Negative impact of meaning in life on death anxiety

In the field of medical psychology, patients’ perception of meaning in life is closely related to their willingness to continue living, especially for patients who lack a sense of meaning in life, they may give up the effort to seek treatment (91). Meaning in life is considered a key psychological factor affecting individuals’ willingness to survive (92). This study further explores the impact of meaning in life on death anxiety in breast cancer patients. The results show that meaning in life is significantly negatively correlated with death anxiety in breast cancer patients, that is, the higher the sense of meaning in life, the lower the level of death anxiety in patients. This finding is consistent with previous studies, indicating that meaning in life can not only help patients better cope with the psychological pressure brought by the disease but also alleviate their fear of death by providing psychological support and goal orientation. In addition, this study also found that meaning in life alleviates death anxiety by enhancing patients’ expectations for positive life experiences, prompting them to complete unfinished business and achieve personal goals. This expectation for positive life experiences helps to alleviate death anxiety. In the study of King and Hicks (93), although the level of meaning in life was explored, the specific mechanism of how meaning in life affects death anxiety was not deeply analyzed. This study fills this gap, provides theoretical support for understanding the psychological experience of breast cancer patients, and offers feasible strategies for reducing patients’ death anxiety in clinical practice. For example, psychological interventions to enhance patients’ self-esteem and sense of meaning in life have been proven to alleviate death anxiety. Moreover, family support and positive psychotherapy have been proven to play an important role in enhancing the sense of meaning in life.

### 4.3 Mediating role of meaning in life

Meaning in life is an individual’s subjective experience of the value and purpose of their own existence, providing a sense of direction and purpose in life (27, 94). For breast cancer patients, meaning in life not only enhances their compliance with treatment but also inspires their hope for cure and expectations for the future (95). This positive psychological state helps patients better cope with the challenges brought by the disease, reducing fear and anxiety about death (96). Family support, as an important social resource for breast cancer patients, its quality and quantity are largely affected by patients’ perception of meaning in life (97). When patients have a positive perception of life, they are more inclined to seek family support, and this seeking and cherishing, in turn, strengthens the intensity and quality of family support. The enhancement of family support, by providing emotional comfort, practical help, and information support, helps alleviate patients’ death anxiety, making them feel more secure and socially connected. However, meaning in life does not exist in isolation; it interacts with family support and death anxiety. The increase in family support can strengthen patients’ positive perception of meaning in life, and the positive perception of meaning in life can further promote the deepening of family support. This interaction constitutes a positive cycle, helping to build a more positive psychological environment, thereby reducing death anxiety. The mediating role of meaning in life is also reflected in its impact on patients’ coping strategies. Patients with a strong sense of meaning in life are more likely to adopt proactive coping strategies, such as actively seeking treatment information, participating in the decision-making process, and adjusting their lifestyle. These proactive coping strategies not only help improve treatment outcomes but also effectively reduce death anxiety. These proactive coping strategies have been shown in empirical studies not only to improve treatment outcomes, but also to be effective in reducing death anxiety. For example, Taber et al. (98) found in a randomized controlled trial that breast cancer patients who participated in an active coping intervention exhibited lower anxiety levels and higher quality of life during treatment. In addition, Lueger-Schuster et al. (99) longitudinal studies that active coping strategies are strongly associated with better mental health outcomes demonstrated through. In clinical practice, understanding and applying the mediating role of meaning in life is of great value for the comprehensive treatment and psychological intervention of breast cancer patients. Through psychological intervention measures, such as meaning in life therapy, patients can be helped to build and strengthen meaning in life, thereby promoting the provision and acceptance of family support, and ultimately achieving the goal of reducing death anxiety.

### 4.4 Practical significance and clinical role

Meaning in life, as a psychological resource, provides psychological resilience for breast cancer patients facing the threat of disease and death, inspires the patients’ will to survive, and enhances their compliance with treatment, thereby improving treatment outcomes to a certain extent (100). Family support plays a crucial role in this process, providing emotional comfort to patients and possibly reducing their physiological and psychological burdens through practical actions such as companionship, economic support, and daily care. Companionship refers to the spiritual presence and emotional support between family members and the patient. This support includes not only physical, such as accompanying patients to the doctor, sharing the same room, etc., but also psychological companionship (such as listening, encouragement, and emotional empathy). Studies have shown that companionship can significantly improve the mental health of breast cancer patients and reduce their death anxiety and depression (17). Further analysis showed that companionship indirectly alleviated death anxiety by enhancing patients’ sense of meaning in life. This finding is consistent with Thoits (101), that companionship, as a form of emotional support, can help patients find meaning in life amid disease challenges, thereby enhancing psychological resilience. However, the effect of family support is not directly on the patients’ mental health but is realized by enhancing patients’ perception of meaning in life. Meaning in life plays a mediating role in this process. When family members affirm and encourage the patients’ meaning in life, patients are more likely to feel their own value and the necessity of treatment. This affirmation and encouragement can be transformed into psychological resilience for patients facing disease challenges, significantly reducing their fear and anxiety about death. Specifically, family support indirectly reduces the level of death anxiety by enhancing patients’ perception of meaning in life, indicating that meaning in life plays an important mediating role in this process. This finding provides theoretical support for family-centered nursing approach and further improves its application strategy in psychological support of breast cancer patients. Family members can help patients access disease-related information, such as treatment options, medication side effects, or recovery resources. This information support can help patients better understand their condition and enhance their sense of control over their treatment. In addition, family members can help patients weigh the pros and cons of treatment by participating in the patient’s decision-making process, thereby improving the patient’s treatment compliance.

In clinical practice, the findings of this study have important application value. First, it emphasizes the importance of psychological intervention in the treatment of breast cancer. Clinical doctors and psychotherapists can design targeted psychological intervention plans to enhance patients’ perception and affirmation of meaning in life, enhance the effect of family support, and thereby reduce patients’ death anxiety. Secondly, this finding also provides guidance for families to help family members better understand and support the psychological needs of patients. Through daily communication, shared activities, and emotional support, family members can strengthen patients’ sense of meaning in life, thereby promoting patients’ mental health at the family level. More importantly, the results of this study have direct implications for healthcare providers. For clinicians, this study highlights the need to consider both the patient’s psychological state and the family support system when making treatment plans. Physicians can provide patients with more comprehensive treatment services through collaboration with mental health professionals, such as during chemotherapy or radiotherapy, combining with the patient’s psychological assessment report to develop an individualized psychological support plan. In addition, clinicians can also assess the psychological state of patients and their families, identify patients with high death anxiety or low sense of meaning in life in time, and provide them with more targeted interventions. For nurses, nurses can help patients relieve anxiety and depression by providing emotional support and psychological counseling. For example, nurses can take the initiative to provide patients with discussions about meaning and values in life and help patients find positive meaning in their illness. This patient-centered nursing model can not only improve the treatment compliance of patients, but also significantly improve the quality of life of patients. In addition, nurses can also help family members better understand the psychological needs of patients by communicating with patients and their families, so as to enhance the effect of family support. Finally, the results of this study provide references for optimizing the allocation of medical resources and improving the service process. For example, medical institutions can provide patients with comprehensive psychological support by increasing investment in mental health services, setting up special psychological support departments, or introducing professional psychological counseling teams.

However, there may be significant differences in family support, perception of meaning in life, and expression of death anxiety across cultures. For example, in collectivist cultures, family support is often more collectivist and interdependent, and family members may provide more practical help and emotional support during illness; However, in the individualist culture, patients may be more inclined to cope with the disease independently, and the form of family support may focus more on emotional support than direct life help. This cultural difference may affect the mediating effect of family support on the perception of meaning in life and thus the level of death anxiety. In addition, the attitude and interpretation of the meaning of death in different cultures (such as religious beliefs, cultural values, etc.) may also lead to differences in the path of the perception of the meaning of life and the expression of death anxiety. In response to these cultural differences, the psychological intervention strategies and clinical application of the results of this study may need to be adjusted according to the specific cultural context. For example, in collectivist cultures, family support training may require a greater emphasis on treatment planning that involves the family together; However, in the individualist culture, psychological intervention may focus more on the construction of personal meaning and the improvement of self-efficacy.

### 4.5 Limitations

Despite the above research findings, we must acknowledge that this study still has many limitations. First, as the first study to link family support and death anxiety, this study only included the mediating variable (meaning in life) and did not further explore whether there is a moderating mechanism between family support and death anxiety. Future research can further explore whether there are other moderating variables in the effect of family support on death anxiety, such as psychological resilience, personality traits, or socioeconomic status of patients. Through in-depth analysis of these potential regulatory mechanisms, the impact path of family support on death anxiety can be more fully understood. Secondly, the study data were mainly collected through the form of self-report questionnaires, which may make our study data more subjective and subject to self-report bias. This deviation may lead to some differences between the actual measurement results and the real situation, and affect the objectivity of the research conclusions. To mitigate the impact of self-report bias, future research can adopt a mixed-methods approach, combining quantitative data with qualitative data, and obtain more comprehensive and in-depth research results through interviews or case studies. In addition, the research data came from a tertiary hospital in China, which is highly regional and cannot avoid the impact of regional development differences. Future studies can expand the sample scope, verify across regions and cultures, and increase the external validity and universality of the research results. This study used a cross-sectional study design, and future longitudinal studies are suggested to track changes in psychological status of breast cancer patients at different time points. Longitudinal studies can help us better understand the dynamics of family support and sense of meaning in life during disease progression and its long-term impact on death anxiety. In addition, longitudinal studies can also provide more informed recommendations on the time window and persistence of interventions, helping clinicians to develop more targeted time planning. Third, we must acknowledge that the failure to include quality of life in this article is an important limitation. Despite the fact that quality of life is a key outcome variable, able to reflect the mental and physical health of patients, we failed to incorporate relevant assessment scales in our study design. The main reasons include: First, our study focused on family support, sense of meaning in life and its impact on death anxiety, and the correlation between quality of life and various variables has been verified in previous studies. Therefore, we have not further explored the relationship between each variable and life. At the same time, although quality-of-life measures such as the EORTC QLQ-C30 or FACT-B are short and easy to apply (102, 103), but in the study design, we prioritized variables that were directly related to the primary study hypothesis. Future research can make up for this deficiency by including the evaluation of quality of life, so as to more comprehensively understand the impact of family support and sense of meaning in life on the comprehensive rehabilitation of patients. Finally, families provide a variety of assistance and support to individuals, and this study exclusively assessed the relationship between family support (as measured by the scale) and patients’ death anxiety. Future research could encompass objective indicators such as family members and family structure.

## 5 Conclusion

Under the guidance of family support theory and meaning therapy theory, this study constructed a mediating model with meaning in life as the mediating variable and found that family support and meaning in life are positively and significantly correlated, family support and death anxiety are negatively and significantly correlated, and meaning in life and death anxiety are negatively and significantly correlated. We also verified that meaning in life has a complete mediating effect between family support and death anxiety. In clinical practice, medical workers can encourage patients to seek family support from their families to enhance their psychological resilience and sense of meaning in life, thereby reducing patients’ fear and death anxiety and improving their quality of life.

## Data Availability

The original contributions presented in the study are included in the article/supplementary material, further inquiries can be directed to the corresponding author.
